# Supplementary material for: Transforming Health Care Delivery towards Value-Based Health Care in Germany: A Delphi Survey among Stakeholders
Source: Healthcare (Basel). 2023 Apr 20;11(8):1187. doi: 10.3390/healthcare11081187 (PMC10138274; doi:10.3390/healthcare11081187)
Supplement: Supplementary file 1 [file healthcare-11-01187-s001.zip › healthcare-2301553-supplementary.pdf]

Supplementary Materials

Table S1. Assessment of steps and activities regarding relevance and feasibility after round 1 of the Delphi survey.

	Items*	Distribution				Central tendency			Consensus	
		a	sa	sd	d	Mean	SD	Median	Direction	Level
		in %				4-level Likert scale				
	**1: Measurement of treatment outcomes and costs for every patient**									
Relevance	Data on outcomes tangible and important to patients	54	36	7	3	1,59	0,75	1	+	high
	Outcome data collection indication-specific	43	38	15	4	1,81	0,85	2	+	high
	Outcome data collection standardized	59	29	6	6	1,59	0,85	1	+	high
	Outcome data collection integrated into daily care	54	28	15	3	1,66	0,84	1	+	high
	Outcome data collection with valid instruments	57	38	3	1	1,49	0,63	1	+	high
	Outcome data on short- and long-term effects of treatment course	59	35	6	0	1,47	0,61	1	+	high
	All providers publish outcome data	40	31	19	10	2,00	1,01	2	+	moderate
	Patient-individual collection of financial resources	28	31	29	12	2,25	1,00	2		dissent
	Evaluation of expended financial resources by clinical and administrative staff	37	29	24	10	2,07	1,01	2	+	low
	Regular team meetings on outcome data	71	26	3	0	1,32	0,53	1	+	high
	Regular team meetings on expended resources	44	29	19	7	1,90	0,96	2	+	moderate
Feasibility	Data on outcomes tangible and important to patients	33	43	19	4	1,94	0,84	2	+	moderate
	Outcome data collection indication-specific	32	50	12	6	1,91	0,82	2	+	high
	Outcome data collection standardized	26	40	28	6	2,13	0,88	2	+	low
	Outcome data collection integrated into daily care	16	19	51	13	2,62	0,91	3	-	low
	Outcome data collection with valid instruments	15	46	32	7	2,32	0,82	2	+	low
	Outcome data on short- and long-term effects of treatment course	13	40	35	12	2,46	0,87	2		dissent
	All providers publish outcome data	15	31	32	22	2,62	0,99	3		dissent
	Patient-individual collection of financial resources	15	32	32	21	2,59	0,98	3		dissent
	Evaluation of expended financial resources by clinical and administrative staff	9	35	38	18	2,65	0,88	3		dissent
	Regular team meetings on outcome data	25	37	31	7	2,21	0,91	2	+	low
	Regular team meetings on expended resources	16	32	37	15	2,50	0,94	3		dissent
	Characteristics of institution responsible for determination, collection and evaluation of treatment outcomes and costs									
Relevance	Independent / neutral / free from conflicts of interest	84	13	3	0	1,19	0,47	1	+	high
	Legitimate members	40	35	18	7	1,93	0,94	2	+	moderate
	Legally defined tasks	43	41	16	0	1,74	0,73	2	+	high
	Equal representation (e.g. payers, service providers, patients)	54	26	9	10	1,75	1,00	1	+	high
	Democratically elected	18	29	32	21	2,56	1,01	3		dissent
	Interdisciplinary	69	26	4	0	1,35	0,57	1	+	high

	Scientific and methodological expertise	59	38	3	0	1,44	0,56	1	+	high
	Scientific-clinical expertise	66	31	1	1	1,38	0,60	1	+	high
Feasibility	Independent / neutral / free from conflicts of interest	25	32	37	6	2,24	0,90	2		dissent
	Legitimate members	32	49	15	4	1,91	0,81	2	+	high
	Legally defined tasks	32	54	13	0	1,81	0,65	2	+	high
	Equal representation (e.g. payers, service providers, patients)	29	46	19	6	2,01	0,86	2	+	moderate
	Democratically elected	18	28	34	21	2,57	1,01	3		dissent
	Interdisciplinary	38	49	9	4	1,79	0,78	2	+	high
	Scientific and methodological expertise	40	49	12	0	1,72	0,67	2	+	high
	Scientific-clinical expertise	43	50	7	0	1,65	0,62	2	+	high
	**2: Organization of care in integrated care facilities & networks**									
Relevance	Multidisciplinary treatment (outpatient, inpatient & rehabilitative services)	80	18	2	0	1,22	0,45	1	+	high
	Indication-specific health care organization	50	30	13	7	1,77	0,93	1,5	+	high
	Health care as joint responsibility of multidisciplinary treatment team	68	20	8	3	1,47	0,79	1	+	high
	Health care planned from the outset	58	35	3	3	1,52	0,72	1	+	high
	Common management structure within a care network	37	33	22	8	2,02	0,97	2	+	moderate
	Common scheduling system within a care network	40	52	7	2	1,70	0,67	2	+	high
	Joint cross-sector payment within a care network	45	32	15	8	1,87	0,96	2	+	moderate
	Management by one team leader per patient within a care network	35	37	22	7	2,00	0,92	2	+	moderate
	Health care for each indication at one location	10	20	48	22	2,82	0,89	3	-	moderate
Feasibility	Multidisciplinary treatment (outpatient, inpatient & rehabilitative services)	18	37	40	5	2,32	0,83	2		dissent
	Indication-specific health care organization	18	43	33	5	2,25	0,82	2	+	low
	Health care as joint responsibility of multidisciplinary treatment team	18	32	32	18	2,50	1,00	2,5		dissent
	Health care planned from the outset	10	35	40	15	2,60	0,87	3		dissent
	Common management structure within a care network	15	30	35	20	2,60	0,98	3		dissent
	Common scheduling system within a care network	25	32	28	15	2,33	1,02	2		dissent
	Joint cross-sector payment within a care network	12	25	42	22	2,73	0,94	3	-	low
	Management by one team leader per patient within a care network	10	30	45	15	2,65	0,86	3	-	low
	Health care for each indication at one location	5	28	43	23	2,85	0,84	3	-	low
	**3: Organization of integrated service provision between facilities**									
Relevance	Specific service offering of each care institution	46	39	8	7	1,76	0,88	2	+	high
	Disease-specific interdisciplinary providers with high treatment volume for scheduled or complex treatments	61	36	3	0	1,42	0,56	1	+	high
	Routine care provision at less costly sites	54	32	8	5	1,64	0,85	1	+	high
	Coordinating institution for cooperation between care institutions	37	22	32	8	2,12	1,02	2		dissent

Feasibility	Specific service offering of each care institution	22	34	34	10	2,32	0,94	2		dissent
	Disease-specific interdisciplinary providers with high treatment volume for scheduled or complex treatments	29	47	19	5	2,00	0,83	2	+	moderate
	Routine care provision at less costly sites	32	34	24	10	2,12	0,98	2	+	low
	Coordinating institution for cooperation between care institutions	15	25	39	20	2,64	0,98	3		dissent
	**4: Geographic expansion of excellent forms of care**									
Relevance	Expanding excellent forms of care rather than the catchment area	29	55	13	4	1,91	0,75	2	+	high
	Cooperations in the form of care networks	66	30	4	0	1,38	0,56	1	+	high
	Care institutions responsible for expansion of collaboration	23	27	36	14	2,41	1,01	2,5		dissent
	Rotation of individual employees between participating care facilities	39	38	18	5	1,89	0,89	2	+	moderate
Feasibility	Expanding excellent forms of care rather than the catchment area	14	48	30	7	2,30	0,81	2	+	low
	Cooperations in the form of care networks	18	41	39	2	2,25	0,77	2		dissent
	Care institutions responsible for expansion of collaboration	16	32	39	13	2,48	0,91	3		dissent
	Rotation of individual employees between participating care facilities	14	27	39	20	2,64	0,96	3		dissent
	**5: Common remuneration of all treatment steps**									
Relevance	Inter-sectoral, risk-adjusted joint budget provided to a care network	22	41	24	13	2,28	0,96	2	+	low
	Inter-sectoral joint budget for indications with multidisciplinary treatment needs	30	39	17	15	2,17	1,02	2	+	low
	Annually adjusted inter-sectoral joint budget	39	37	11	13	1,98	1,02	2	+	moderate
	Inter-sectoral joint budget based on treatment outcomes	17	48	9	26	2,44	1,06	2	+	low
	No reimbursement of costs for preventable events	17	24	35	24	2,67	1,03	3		dissent
	Additional reimbursement of costs for unavoidable events	54	30	11	6	1,69	0,89	1	+	high
Feasibility	Inter-sectoral, risk-adjusted joint budget provided to a care network	15	33	35	17	2,54	0,95	3		dissent
	Inter-sectoral joint budget for indications with multidisciplinary treatment needs	19	46	22	13	2,30	0,92	2	+	low
	Annually adjusted inter-sectoral joint budget	30	35	26	9	2,15	0,96	2	+	low
	Inter-sectoral joint budget based on treatment outcomes	7	20	41	31	2,96	0,91	3	-	moderate
	No reimbursement of costs for preventable events	19	28	33	20	2,56	1,02	3		dissent
	Additional reimbursement of costs for unavoidable events	41	26	20	13	2,06	1,07	2	+	low
	**6: Establishment of an information technology**									
Relevance	Digital patient record for each patient	89	9	2	0	1,13	0,39	1	+	high
	Data relevant to care covers the entire course of treatment	87	11	0	2	1,17	0,51	1	+	high
	Standardized structure of digital patient record	91	8	2	0	1,11	0,38	1	+	high
	Digital patient record accessible to all providers involved in care	83	13	4	0	1,21	0,49	1	+	high
	Digital patient record as intelligent system with disease-specific recommendations	64	25	8	4	1,51	0,80	1	+	high

Feasibility	Digital patient record for each patient	40	34	26	0	1,87	0,81	2	+	moderate
	Data relevant to care covers the entire course of treatment	36	34	28	2	1,96	0,85	2	+	low
	Standardized structure of digital patient record	38	36	21	6	1,94	0,91	2	+	moderate
	Digital patient record accessible to all providers involved in care	38	36	25	2	1,91	0,84	2	+	moderate
	Digital patient record as intelligent system with disease-specific recommendations	26	42	21	11	2,17	0,96	2	+	low
	**7: Improve transparency**									
Relevance	Clear communication of responsibilities in macro-level decisions	61	29	10	0	1,49	0,67	1	+	high
	Clear communication of decision criteria in macro-level decisions	75	25	0	0	1,25	0,44	1	+	high
	Clear communication of data bases in macro-level decisions	71	24	6	0	1,35	0,59	1	+	high
	Clear communication of decision-making bodies in macro-level decisions	65	27	8	0	1,43	0,64	1	+	high
	Clear communication of conflicts of interest in macro-level decisions	69	27	4	0	1,35	0,56	1	+	high
	Patient-comprehensible communication in micro-level decisions	82	16	2	0	1,20	0,45	1	+	high
	Presentation of different treatment options to patients in micro-level decisions	73	25	2	0	1,29	0,50	1	+	high
	Structured shared decision making in micro-level decisions	76	20	4	0	1,27	0,53	1	+	high
	Structured advice on self-management and health promotion in micro-level decisions	75	20	6	0	1,31	0,58	1	+	high
Feasibility	Clear communication of responsibilities in macro-level decisions	35	41	22	2	1,90	0,81	2	+	moderate
	Clear communication of decision criteria in macro-level decisions	37	39	22	2	1,88	0,82	2	+	moderate
	Clear communication of data bases in macro-level decisions	35	51	12	2	1,80	0,72	2	+	high
	Clear communication of decision-making bodies in macro-level decisions	45	41	12	2	1,71	0,76	2	+	high
	Clear communication of conflicts of interest in macro-level decisions	43	27	24	6	1,92	0,96	2	+	moderate
	Patient-comprehensible communication in micro-level decisions	47	43	10	0	1,63	0,66	2	+	high
	Presentation of different treatment options to patients in micro-level decisions	43	43	14	0	1,71	0,70	2	+	high
	Structured shared decision making in micro-level decisions	41	43	16	0	1,75	0,72	2	+	high
	Structured advice on self-management and health promotion in micro-level decisions	41	45	14	0	1,73	0,70	2	+	high

*Displayed item descriptions represent short versions, for full length see Table S3.

a = agree, sa = somewhat agree, sd = somewhat disagree, d = disagree

Table S2: Assessment of steps and activities regarding relevance and feasibility after round 2 of the Delphi survey.

		Distribution				Central tendency			Consensus		Stability of responses	
	Items*	a	sa	sd	d	Mean	SD	Median	Direction	Level	Chi^2	Correlation
		in %				4-level Likert scale						
	**1: Measurement of treatment outcomes and costs for every patient**											
Relevance	Patient-individual collection of financial resources	31	42	15	12	2,08	0,97	2	+	moderate	3,64	0,24
Relevance	Evaluation of expended financial resources by clinical and administrative staff	42	37	15	6	1,85	0,89	2	+	moderate	2,39	0,20
Feasibility	Outcome data collection standardized	52	29	15	4	1,71	0,87	1	+	high	8,39	0,36
Feasibility	Outcome data collection integrated into daily care	52	29	15	4	1,71	0,87	1	+	high	26,63	0,60
Feasibility	Outcome data collection with valid instruments	52	40	6	2	1,58	0,70	1	+	high	25,15	0,59
Feasibility	Outcome data on short- and long-term effects of treatment course	38	48	10	4	1,79	0,78	2	+	high	18,49	0,52
Feasibility	All providers publish outcome data	37	31	27	6	2,02	0,94	2	+	low	11,31	0,42
Feasibility	Patient-individual collection of financial resources	25	38	23	13	2,25	0,99	2	+	low	3,69	0,24
Feasibility	Evaluation of expended financial resources by clinical and administrative staff	38	29	25	8	2,02	0,98	2	+	low	16,10	0,49
Feasibility	Regular team meetings on outcome data	42	44	12	2	1,73	0,74	2	+	high	9,76	0,39
Feasibility	Regular team meetings on expended resources	33	35	33	0	2,00	0,82	2	+	low	11,28	0,41
	Characteristics of institution responsible for determination, collection and evaluation of treatment outcomes and costs											
Relevance	Democratically elected	33	25	27	15	2,25	1,08	2		dissent	3,69	0,24
Feasibility	Independent / neutral / free from conflicts of interest	52	29	19	0	1,67	0,79	1	+	high	12,11	0,43
Feasibility	Democratically elected	25	35	27	13	2,29	1,00	2		dissent	2,50	0,20
	**2: Organization of care in integrated care facilities & networks**											
Feasibility	Multidisciplinary treatment (outpatient, inpatient & rehabilitative services)	50	31	17	2	1,71	0,82	1,5	+	high	13,99	0,48
Feasibility	Indication-specific health care organization	44	42	13	2	1,73	0,76	2	+	high	11,25	0,43
Feasibility	Health care as joint responsibility of multidisciplinary treatment team	44	31	21	4	1,85	0,90	2	+	moderate	11,43	0,44
Feasibility	Health care planned from the outset	44	44	10	2	1,71	0,74	2	+	high	26,17	0,62
Feasibility	Common management structure within a care network	29	31	27	13	2,23	1,02	2	+	low	3,96	0,27
Feasibility	Common scheduling system within a care network	40	42	10	8	1,88	0,91	2	+	high	7,73	0,37
Feasibility	Joint cross-sector payment within a care network	38	19	31	13	2,19	1,08	2		dissent	10,21	0,42
Feasibility	Management by one team leader per patient within a care network	33	42	19	6	1,98	0,89	2	+	moderate	15,51	0,50
Feasibility	Health care for each indication at one location	15	29	44	13	2,54	0,90	3		dissent	4,34	0,28

	**3: Organization of integrated service provision between facilities**											
Relevance	Coordinating institution for cooperation between care institutions	26	60	11	4	1,94	0,73	2	+	high	16,74	0,52
Feasibility	Specific service offering of each care institution	23	57	19	0	1,96	0,66	2	+	high	10,15	0,42
	Routine care provision at less costly sites	49	45	6	0	1,57	0,62	2	+	high	12,32	0,46
	Coordinating institution for cooperation between care institutions	23	45	21	11	2,19	0,92	2	+	low	7,95	0,37
	**4: Geographic expansion of excellent forms of care**											
Relevance	Care institutions responsible for expansion of collaboration	30	34	30	6	2,13	0,92	2	+	low	2,63	0,22
Feasibility	Expanding excellent forms of care rather than the catchment area	21	45	30	4	2,17	0,82	2	+	low	1,15	0,15
	Cooperations in the form of care networks	49	36	11	4	1,70	0,83	2	+	high	16,40	0,52
	Care institutions responsible for expansion of collaboration	26	28	38	9	2,30	0,95	2		dissent	1,68	0,18
	Rotation of individual employees between participating care facilities	34	28	34	4	2,09	0,93	2	+	low	9,27	0,41
	**5: Common remuneration of all treatment steps**											
Relevance	Inter-sectoral, risk-adjusted joint budget provided to a care network	20	53	24	2	2,09	0,73	2	+	moderate	4,40	0,29
	Inter-sectoral joint budget for indications with multidisciplinary treatment needs	31	44	22	2	1,96	0,80	2	+	moderate	4,88	0,31
	Inter-sectoral joint budget based on treatment outcomes	27	29	42	2	2,20	0,87	2		dissent	23,57	0,62
	No reimbursement of costs for preventable events	13	40	36	11	2,44	0,87	2		dissent	4,44	0,29
Feasibility	Inter-sectoral, risk-adjusted joint budget provided to a care network	18	33	38	11	2,42	0,92	2		dissent	0,71	0,12
	Inter-sectoral joint budget for indications with multidisciplinary treatment needs	31	51	16	2	1,89	0,75	2	+	high	5,80	0,33
	Annually adjusted inter-sectoral joint budget	36	31	29	4	2,02	0,92	2	+	low	1,27	0,16
	No reimbursement of costs for preventable events	18	33	33	16	2,47	0,97	2		dissent	0,57	0,11
	Additional reimbursement of costs for unavoidable events	47	38	13	2	1,71	0,79	2	+	high	5,51	0,32
	**6: Establishment of an information technology**											
Feasibility	Data relevant to care covers the entire course of treatment	59	25	14	2	1,59	0,82	1	+	high	5,85	0,34
	Digital patient record as intelligent system with disease-specific recommendations	52	34	9	5	1,66	0,83	1	+	high	8,01	0,39

*Displayed item descriptions represent short versions, for full length see Table S3.
a = agree, sa = somewhat agree, sd = somewhat disagree, d = disagree

Table S3: Survey questions in German, English and short English form. Participants were asked to rate the relevance as well as feasibility of each statement.

No.	Item in German	Item in English	Short English form
**1.1**	Die Datenerhebung zu Behandlungsergebnissen sollte hauptsächlich für Patient*innen spürbare und wichtige Behandlungsergebnisse (z.B. Schmerzen, Funktionsfähigkeit, Rezidiv) zur Beurteilung der tatsächlichen Auswirkungen der gesundheitlichen Versorgung, umfassen.	Data collection on treatment outcomes should primarily include treatment outcomes that are tangible and important to patients (e.g., pain, functional capacity, recurrence) to assess the true impact of health care.	Data on outcomes tangible and important to patients
**1.2**	Die Datenerhebung zu Behandlungsergebnissen sollte immer indikationsspezifisch erfolgen.	Data collection on treatment outcomes should always be indication specific.	Outcome data collection indication-specific
**1.3**	Die Datenerhebung zu Behandlungsergebnissen sollte immer standardisiert erfolgen (d.h. mit festgelegten Verfahren zu festgelegten Zeitpunkten).	Data collection on treatment outcomes should always be standardized (i.e., using established procedures at established time points).	Outcome data collection standardized
**1.4**	Die Datenerhebung zu Behandlungsergebnissen sollte bei allen Patient*innen in den Versorgungsalltag integriert werden.	Data collection on treatment outcomes should be integrated into the daily care of all patients.	Outcome data collection integrated into daily care
**1.5**	Die Datenerhebung zu Behandlungsergebnissen sollte nur mit validen Instrumenten durchgeführt werden.	Data collection on treatment outcomes should be conducted only with valid instruments.	Outcome data collection with valid instruments
**1.6**	Die Daten zu Behandlungsergebnissen sollten kurz- und langfristige Auswirkungen der gesundheitlichen Versorgung wiederholt über den gesamten Behandlungsverlauf abbilden.	Treatment outcome data should reflect short- and long-term effects of health care repeatedly throughout the course of treatment.	Outcome data on short- and long-term effects of treatment course
**1.7**	Die Daten zu Behandlungsergebnissen sollten von jeder Versorgungseinrichtung veröffentlicht werden.	Treatment outcome data should be published by each care provider.	All providers publish outcome data
**1.8**	Die aufgewendeten finanziellen Ressourcen der Versorgung sollten für alle Patient*innen einzeln erhoben werden.	The financial resources spent on care should be collected individually for all patients.	Patient-individual collection of financial resources expended
**1.9**	Die aufgewendeten finanziellen Ressourcen der Versorgung sollten gemeinsam von klinischem und administrativem Personal bewertet werden.	Financial resources of care expended should be evaluated jointly by clinical and administrative staff.	Evaluation of expended financial resources by clinical and administrative staff
**1.10**	Es sollten regelmäßige Teambesprechungen zu den Daten über Behandlungsergebnisse stattfinden.	There should be regular team meetings on treatment outcome data.	Regular team meetings on outcome data
**1.11**	Es sollten regelmäßige Teambesprechungen zu den Daten über aufgewendete Ressourcen stattfinden.	There should be regular team meetings regarding data on resources expended.	Regular team meetings on expended resources
**1.12**	Unabhängig / neutral / frei von Interessenkonflikten	Independent / neutral / free from conflicts of interest	Independent / neutral / free from conflicts of interest

**1.13**	Gesetzlich legitimierte Mitglieder	Legitimate members	Legitimate members
**1.14**	Gesetzlich definierte Aufgaben	Legally defined tasks	Legally defined tasks
**1.15**	Paritätisch besetzt (z.B. Kostenträger, Leistungserbringende, Patient*innen)	Equal representation (e.g. payers, service providers, patients)	Equal representation (e.g. payers, service providers, patients)
**1.16**	Demokratisch gewählt	Democratically elected	Democratically elected
**1.17**	Interdisziplinär (z.B. verschiedene fachlich relevante Leistungserbringende für die Fragestellung, Wissenschaftler*innen)	Interdisciplinary (e.g. different relevant service providers for the question, scientists)	Interdisciplinary
**1.18**	Wissenschaftlich-methodische Expertise	Scientific and methodological expertise	Scientific and methodological expertise
**1.19**	Wissenschaftlich-klinische Expertise	Scientific-clinical expertise	Scientific-clinical expertise
**2.1**	Die gesundheitliche Versorgung durch ein multidisziplinäres Behandlungsteam sollte ambulante, stationäre und rehabilitative Versorgungsleistungen umfassen.	Health care provided by a multidisciplinary treatment team should include outpatient, inpatient, and rehabilitative care services.	Multidisziplinary treatment (outpatient, inpatient & rehabilitative services)
**2.2**	Die gesundheitliche Versorgung sollte an Hand von indikationsspezifischen Behandlungsverläufen organisiert werden, d.h. nicht auf Grundlage einzelner Versorgungsleistungen.	Health care should be organized on the basis of indication-specific treatment pathways, i.e. not on the basis of individual care services.	Indication-specific health care organization
**2.3**	Die gesundheitliche Versorgung sollte gemeinsam durch das multidisziplinäre Behandlungsteam verantwortet werden.	Health care should be the joint responsibility of the multidisciplinary treatment team.	Health care as joint responsibility of multidisciplinary treatment team
**2.4**	Die gesundheitliche Versorgung sollte von vornherein umfassend geplant werden und u.a. präventive, diagnostische, therapeutische, rehabilitative oder palliative Leistungen berücksichtigen.	Health care should be planned comprehensively from the outset, taking into account preventive, diagnostic, therapeutic, rehabilitative or palliative services, among others.	Health care planned from the outset
**2.5**	Die gesundheitliche Versorgung sollte innerhalb eines Versorgungsnetzwerkes durch eine gemeinsame Verwaltungsstruktur umgesetzt werden.	Health care should be implemented within a care network through a common management structure.	Common management structure within a care network
**2.6**	Die gesundheitliche Versorgung sollte innerhalb eines Versorgungsnetzwerkes durch eine gemeinsame Terminplanungsstruktur umgesetzt werden.	Health care should be implemented within a care network through a common scheduling structure.	Common scheduling system within a care network
**2.7**	Die gesundheitliche Versorgung sollte innerhalb eines Versorgungsnetzwerkes durch eine gemeinsame sektorenübergreifenden Vergütung finanziert werden.	Health care should be financed within a care network through joint cross-sector payment.	Joint cross-sector payment within a care network
**2.8**	Die gesundheitliche Versorgung sollte innerhalb eines Versorgungsnetzwerkes von einer Teamleitung je Patient*in betreut werden.	Health care should be managed by one team leader per patient within a care network.	Management by one team leader per patient within a care network
**2.9**	Die gesundheitliche Versorgung sollte innerhalb eines Versorgungsnetzwerkes für jede Indikation möglichst an einem Standort erbracht werden.	Health care should be provided within a care network for each indication at one location, if possible.	Health care for each indication at one location

**3.1**	Jede Versorgungseinrichtung sollte ein spezifisches Leistungsangebot festlegen, d.h. die Versorgung in den ausgewählten Bereichen verbessern und bestimmte Leistungen dafür nicht anbieten.	Each care institution should establish a specific service offering, i.e., improve care in the selected areas and not offer certain services in return.	Specific service offering of each care institution
**3.2**	Planbare oder komplexe Behandlungen (z.B. chirurgische Eingriffe bei Prostatakrebs) sollten von einer begrenzten Anzahl erkrankungsspezifischer interdisziplinärer Versorgungseinrichtungen mit einem hohen Behandlungsvolumen umgesetzt werden.	Scheduled or complex treatments (e.g., surgical procedures for prostate cancer) should be implemented by a limited number of disease-specific interdisciplinary care providers with a high volume of treatment.	Disease-specific interdisciplinary providers with high treatment volume for scheduled or complex treatments
**3.3**	Weniger komplexe Behandlungen und Routinebehandlungen sollten an weniger kostenintensiven Standorten außerhalb von Universitätskliniken erbracht werden.	Less complex treatments and routine care should be provided at less costly sites outside of university hospitals.	Routine care provision at less costly sites
**3.4**	Eine koordinierende Einrichtung sollte die Zusammenarbeit aller beteiligten Versorgungseinrichtungen zentral planen.	A coordinating institution should centrally plan the cooperation of all participating care institutions.	Coordinating institution for cooperation between care institutions
**4.1**	Der Fokus sollte auf der Ausweitung exzellenter Versorgungsformen anstelle der Ausweitung des Einzugsgebiets eines Versorgungsnetzwerks liegen.	The focus should be on expanding excellent forms of care rather than expanding the catchment area of a care network.	Expanding excellent forms of care rather than the catchment area
**4.2**	Zur Förderung exzellenter Versorgung sollten Kooperationen, in Form von Versorgungsnetzwerken, zwischen z.B. niedergelassenen Praxen, Krankenhäusern der Grund- und Regelversorgung sowie überregionalen Kliniken gebildet werden.	In order to promote excellent care, cooperations in the form of care networks should be formed between, for example, private practices, hospitals providing basic and standard care, and supraregional clinics.	Cooperations in the form of care networks
**4.3**	Die Ausweitung von Kooperationen innerhalb der Versorgungsnetzwerke sollte in Eigenverantwortung der beteiligten Versorgungseinrichtungen erfolgen.	The expansion of collaborations within the care networks should be the responsibility of the participating care institutions.	Care institutions responsible for expansion of collaboration
**4.4**	Einzelne Mitarbeitende des Versorgungsnetzwerks sollten regelmäßig zwischen den beteiligten Versorgungseinrichtungen wechseln, um den fachlichen Austausch und Zusammenhalt innerhalb des Netzwerks zu fördern.	Individual employees of the care network should regularly rotate between the participating care facilities in order to promote professional exchange and cohesion within the network.	Rotation of individual employees between participating care facilities
**5.1**	Das sektorenübergreifende gemeinsames Budget sollte risikoadjustiert an ein Versorgungsnetzwerk erfolgen.	The inter-sectoral joint budget should be risk-adjusted to a network of care.	Inter-sectoral, risk-adjusted joint budget to a care network
**5.2**	Das sektorenübergreifende gemeinsames Budget sollte insbesondere für spezifische Indikationen mit multidisziplinärem Behandlungsbedarf (z.B. chronische Erkrankungen) eingeführt werden.	The inter-sectoral joint budget should be introduced in particular for specific indications with multidisciplinary treatment needs (e.g., chronic diseases).	Inter-sectoral joint budget for indications with multidisciplinary treatment needs
**5.3**	Das sektorenübergreifende gemeinsames Budget sollte jährlich angepasst ausgezahlt werden.	The inter-sectoral joint budget should be disbursed on an annually adjusted basis.	Annually adjusted inter-sectoral joint budget

**5.4**	Das sektorenübergreifende gemeinsames Budget sollte sich hauptsächlich nach den erreichten Behandlungsergebnissen der Patient*innen richten, d.h. höhere Vergütungen für bessere Behandlungsergebnisse.	The inter-sectoral joint budget should be based primarily on the treatment outcomes achieved by patients, i.e. higher reimbursements for better treatment outcomes.	Inter-sectoral joint budget based on treatment outcomes
**5.5**	Zusätzliche Behandlungskosten für vermeidbare Ereignisse (z.B. Wundinfektion nach Operation), sollten nicht zusätzlich vergütet werden.	Additional treatment costs for preventable events (e.g., wound infection after surgery), should not be reimbursed additionally.	No reimbursement of costs for preventable events
**5.6**	Zusätzliche Behandlungskosten für unvermeidbare Ereignisse sollten zusätzlich vergütet werden.	Additional treatment costs for unavoidable events should be reimbursed additionally.	Additional reimbursement of costs for unavoidable events
**6.1**	Versorgungsrelevante Daten sollten für jede*n einzelne*n Patient*in in einer digitalen Patient*innenakte erfasst werden.	Data relevant to the provision of care should be recorded in a digital patient record for each individual patient.	Digital patient record for each patient
**6.2**	Versorgungsrelevante Daten sollten den gesamten Behandlungsverlauf umfassen, d.h. relevante Anamnesedaten sowie diagnostische und therapeutische Informationen beinhalten.	Data relevant to care should cover the entire course of treatment, i.e., include relevant medical history data as well as diagnostic and therapeutic information.	Data relevant to care cover the entire course of treatment
**6.3**	Die digitale Patient*innenakte sollte eine standardisierte Datenstruktur und -eingabe haben, um eine einrichtungsübergreifende Zusammenarbeit zu ermöglichen.	The digital patient record should have a standardized data structure and input to enable collaboration across institutions.	Standardized structure of digital patient record
**6.4**	Die digitale Patient*innenakte sollte für alle an der gesundheitlichen Versorgung beteiligten Leistungserbringenden zugänglich sein.	The digital patient record should be accessible to all service providers involved in health care.	Digital patient record accessible to all providers involved in care
**6.5**	Die digitale Patient*innenakte sollte ein intelligentes System sein, welches krankheitsspezifische Empfehlungen gibt (z.B. automatisierter Hinweis auf weitere Diagnostik bei auffälligem Blutbild).	The digital patient file should be an intelligent system that provides disease-specific recommendations (e.g. automated indication of further diagnostics in the case of abnormal blood work).	Digital patient record as intelligent system with disease-specific recommendations
**7.1**	Um Transparenz bei Entscheidungen auf Makroebene (z.B. Krankenhausplanung, Vergütungs- und Erstattungsregelungen) zu verbessern, sollten Verantwortlichkeiten klarer kommuniziert werden.	To improve transparency in macro-level decisions (e.g., hospital planning, payment and reimbursement arrangements), responsibilities should be more clearly communicated.	Clear communication of responsibilities in macro-level decisions
**7.2**	Um Transparenz bei Entscheidungen auf Makroebene (z.B. Krankenhausplanung, Vergütungs- und Erstattungsregelungen) zu verbessern, sollten Entscheidungskriterien klarer kommuniziert werden.	To improve transparency in macro-level decisions (e.g., hospital planning, payment and reimbursement arrangements), decision criteria should be communicated more clearly.	Clear communication of decision criteria in macro-level decisions
**7.3**	Um Transparenz bei Entscheidungen auf Makroebene (z.B. Krankenhausplanung, Vergütungs- und Erstattungsregelungen) zu verbessern, sollten Datengrundlagen klarer kommuniziert werden.	To improve transparency in macro-level decisions (e.g., hospital planning, payment and reimbursement regulations), data bases should be communicated more clearly.	Clear communication of data bases in macro-level decisions

**7.4**	Um Transparenz bei Entscheidungen auf Makroebene (z.B. Krankenhausplanung, Vergütungs- und Erstattungsregelungen) zu verbessern, sollten die Besetzung von Entscheidungsgremien, d.h. die darin vertretenen Interessensgruppen mit ihren Einflussmöglichkeiten, klarer kommuniziert werden.	To improve transparency in macro-level decisions (e.g., hospital planning, remuneration and reimbursement regulations), the composition of decision-making bodies, i.e., the interest groups represented in them with their opportunities to exert influence, should be communicated more clearly.	Clear communication of decision-making bodies in macro-level decisions
**7.5**	Um Transparenz bei Entscheidungen auf Makroebene (z.B. Krankenhausplanung, Vergütungs- und Erstattungsregelungen) zu verbessern, sollten mögliche Interessenskonflikte klarer kommuniziert werden.	To improve transparency in macro-level decisions (e.g., hospital planning, payment and reimbursement regulations), potential conflicts of interest should be communicated more clearly.	Clear communication of conflicts of interest in macro-level decisions
**7.6**	Um Transparenz bei Entscheidungen auf Mikroebene (Interaktion zwischen Patient*innen und Kontaktpersonen in Versorgungseinrichtungen) zu verbessern, sollten individuelle Therapieempfehlungen für Patient*innen verständlicher kommuniziert werden.	In order to improve transparency in micro-level decision-making (interaction between patients and contacts in care facilities), individual therapy recommendations should be communicated in a way that is more comprehensible to patients.	Patient-comprehensible communication in micro-level decisions
**7.7**	Um Transparenz bei Entscheidungen auf Mikroebene (Interaktion zwischen Patient*innen und Kontaktpersonen in Versorgungseinrichtungen) zu verbessern, sollten verschiedenen Behandlungsoptionen für Patient*innen regelhaft aufgezeigt werden.	In order to improve transparency in micro-level decision-making (interaction between patients and contacts in care facilities), different treatment options should be regularly presented to patients.	Presentation of different treatment options to patients in micro-level decisions
**7.8**	Um Transparenz bei Entscheidungen auf Mikroebene (Interaktion zwischen Patient*innen und Kontaktpersonen in Versorgungseinrichtungen) zu verbessern, sollten Patient*innen strukturierter in gemeinsame Behandlungsentscheidungen eingebunden werden.	To improve transparency in micro-level decisions (interaction between patients and contacts in care settings), patients should be involved in shared treatment decisions in a more structured way.	Structured shared decision making in micro-level decisions
**7.9**	Um Transparenz bei Entscheidungen auf Mikroebene (Interaktion zwischen Patient*innen und Kontaktpersonen in Versorgungseinrichtungen) zu verbessern, sollten Patient*innen zu Selbstmanagement und gesundheitsförderlichen Verhaltensweisen strukturierter beraten werden.	To improve transparency in micro-level decision-making (interaction between patients and contacts in care settings), patients should receive more structured advice on self-management and health-promoting behaviors.	Structured advice on self-management and health promotion in micro-level decisions
